# Application of Concomitant Transcranial Direct Current Stimulation (tDCS) and Cognitive Behavioral-Oriented Training (CBT) for Pragmatic Skills Improvement in Young Adults with Autism Spectrum Disorder (ASD): Preliminary Data from a Pilot Study

**DOI:** 10.3390/brainsci15090970

**Published:** 2025-09-10

**Authors:** Lucrezia Arturi, Chiara Scoppola, Assia Riccioni, Martina Siracusano, Luigi Iasevoli, Giulia Civetta, Gianfranco Spalletta, Valentina Fiori, Luigi Mazzone

**Affiliations:** 1 Child Neurology and Psychiatry Unit, Department of Wellbeing of Mental and Neurological, Dental and Sensory Organ Health, Policlinico Tor Vergata Hospital, Viale Oxford 81, 00133 Rome, Italy; 2 Department of Biomedicine and Prevention, University of Rome Tor Vergata, Via Montpellier 1, 00133 Rome, Italy; 3 Multiple Sclerosis Unit, IRCCS Santa Lucia Foundation, Via Ardeatina 306–354, 00179 Rome, Italy; 4 Clinical and Behavioral Neurology, IRCCS Santa Lucia Foundation, Via Ardeatina 306–354, 00179 Rome, Italy; 5 Neuropsychiatry Laboratory, Department of Clinical Neuroscience and Neurorehabilitation, IRCCS Santa Lucia Foundation, Via Ardeatina 306–354, 00179 Rome, Italy; 6 Systems Medicine Department, University of Rome Tor Vergata, Via Montpellier 1, 00133 Rome, Italy

**Keywords:** autism, tDCS, neuromodulation, brain stimulation, social communication, neurodevelopment, language, Broca’s area, brain connectivity

## Abstract

**Objectives:** Individuals with Autism Spectrum Disorder (ASD) exhibit difficulties in the social use of language, regardless of age, cognitive abilities, and symptom severity. The left Broca’s area and adjacent cortex are crucial for socio-pragmatic language, particularly in retrieving and integrating context-dependent words. Neuroimaging studies in ASD have shown hypoactivation of the Broca’s area and an aberrant pattern of functional connectivity between language-related regions, suggesting their potential involvement in socio-communicative deficits. Given the potential of tDCS to modulate brain activity, its application targeting Broca’s areas in addition to psychological intervention may represent a promising approach for enhancing socio-communicative skills in ASD. Thus, this study aims to investigate the effect of concomitant anodal tDCS and cognitive behavioral-oriented training (CBT) on pragmatic and communicative skills in young adults with ASD. **Methods:** A sample of 10 ASD individuals (18–25 years) underwent treatment with both active and sham tDCS targeting the left Broca’s area during concomitant CBT. Each condition was delivered for five consecutive days, and the order of the conditions was blindly randomized. **Results:** Active tDCS significantly improved global communicative and pragmatic abilities compared to sham. A negative correlation was observed between communicative skills improvement and Intelligence Quotient (IQ); no significant association was found between IQ and ASD symptoms’ severity. **Conclusions:** Multisession tDCS targeting the left Broca’s area, combined with CBT, may enhance social language in terms of both production and comprehension of non-literal meanings, supporting Broca’s area as a central neural hub for social language.

## 1. Introduction

Autism Spectrum Disorder (ASD) is a complex neurodevelopmental condition characterized by persistent impairment of social communication and interaction, alongside restricted and repetitive patterns of behavior, interests, or activities [1].

Communication impairment in ASD represents a core aspect of the disorder even if, considering the spectrum, the severity of this deficit is extremely variable. While roughly 25–30% of individuals with ASD are minimally verbal and use single words or sounds to communicate [2], 70% progressively acquire verbal language abilities [3,4,5]. Nevertheless, it is known that conversational and pragmatic aspects of communication—meaning the ability to use language according to the context—remain impaired in autistic individuals, regardless of age, symptom severity level, and cognitive abilities [6,7,8].

Indeed, over the last two decades, research has predominantly focused on assessing and treating socio-pragmatic skills since pragmatics constitutes a core aspect of social interaction, and its impairment significantly impacts the individuals’ quality of life (QoL), representing a significant risk factor for social isolation [8,9,10].

This risk increases during the transition to adulthood when the presence of communicative deficits represents a substantial barrier to the social, occupational, and personal functioning of young adults with ASD [11].

Thus, up to date, the international guidelines on autism treatment recommend psychoeducational, speech, and cognitive-behavioral approaches to improve the socio-communication deficit and pragmatic language impairment [12,13]. In particular, although a positive effect of cognitive-behavioral therapy (CBT) programs on socio-communicative skills has been described, this effect is still limited; therefore, other methods aimed at enhancing communication abilities in this clinical population are being investigated [14].

In the context of neuropsychiatric disorders’ research (including ASD), non-invasive brain stimulation (NIBS) methods, such as transcranial direct current stimulation (tDCS), have been explored as possible therapeutic options to modify pathological neuroplasticity involved in these conditions by targeting function-specific brain areas or structures [15].

Particularly, available data yet highlighted the potential role of NIBS as an add-on method to boost the efficacy of existing psychological or pharmacological treatments for neurological and psychiatric disorders [16,17,18].

From a neurofunctional point of view, fMRI studies focused on the neural basis of language and communication skills in ASD demonstrated that the hypoactivation of Broca’s area and the decreased connectivity between language-related structures of the left hemisphere may represent a crucial factor involved in the atypical communication observed in this clinical population [19,20]. More recently, the results obtained from a study conducted on 618 individuals with ASD in the context of the Autism Brain Imaging Data Exchange-ABIDE [21]—confirmed these previous findings, addressing the hypoactivation of Broca’s area and the altered functional connectivity (FC) between this area and other brain structures involved in verbal processing and social language (left thalamus, precuneus, anterior cingulate cortex, and medial prefrontal cortex) as neurobiological factors underlying communication deficits in ASD.

These findings suggest new pathways for addressing social communication deficits in individuals with ASD through a neuromodulation approach targeting Broca’s area. This approach is supported by a previous study conducted by Marangolo et al. [22] on individuals with communicative impairments due to a neurological condition (chronic aphasia), highlighting the efficacy of the stimulation of Broca’s area for enhancing pragmatic language.

Particularly, the improvement in the use of more contextual utterances (verbs and sentences) after multisession anodal tDCS over Broca’s area, compared to sham and Wernicke’s area stimulation, supports the evidence of Broca’s area as a neural hub involved in pragmatics, more specifically in lexical retrieval and context-dependent word joining [23].

Moreover, a recent fMRI study [24] has shown that during conversation, Broca’s area is engaged not only in combinatorial syntactic and contextual processing but also acts in coordination with brain regions implicated in Theory of Mind (ToM)—such as the medial prefrontal cortex (mPFC) and the middle/superior frontal gyrus—in order to manage the interlocutor’s perspective and plan contextually appropriate communicative acts [6,24].

Despite this evidence, to the best of our knowledge, there are no studies expressly designed to assess the possible efficacy of tDCS targeting neural hubs implied in socio-pragmatic language in the context of autism research. Hence, the present double-blind, randomized, sham-controlled, cross-over pilot study aims at evaluating the effect of concomitant anodal tDCS on left Broca’s and cognitive behavioral-oriented (CBT) training for the improvement of communicative and pragmatic skills in a group of young adults with ASD.

## 2. Materials and Methods

This is a double-blind, randomized, sham-controlled, cross-over pilot study conducted according to the guidelines of the Declaration of Helsinki and approved by the Ethics Committees of the University Hospital Tor Vergata of Rome (#206/21) and Scientific Institute for Research, Hospitalization, and Healthcare (IRCCS) Santa Lucia Foundation (#931). Written informed consent was obtained from all participants and legal holders of custody before the experiment.

### 2.1. Participants

Participants were recruited from the Children and Adolescent Psychiatry Unit of the University Hospital Tor Vergata of Rome. From an initial sample of n = 15 ASD individuals, n = 5 were excluded (n = 4 individuals did not reach the inclusion criteria, and one dropped out of the study after the baseline evaluation).

To be included in the present study, participants were required to have the following: (a) a diagnosis of ASD according to Diagnostic and Statistical Manual of Mental Disorders, Fifth Edition (DSM-5) criteria [25], supported by clinical observation and a structured assessment performed with standardized tools and (b) an age range between 18 and 25 years.

Exclusion criteria were the presence of intellectual disability (IQ equal to or below 70), a severe language impairment, a current or past history of epilepsy, seizures, or head injury, and the presence of any co-occurring neuropsychiatric conditions.

Each participant continued their usual psychological and/or pharmacological treatment in a stable manner for the entire duration of the study.

### 2.2. Experimental Procedure

**Figure 1:**
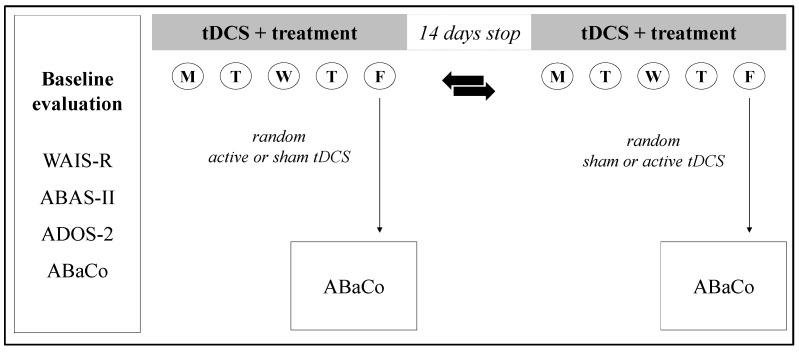
Summary of the study design. Legend: WAIS-R, Weschler Adult Intelligence Scale-Revised; ABAS-II, Adaptive Behavior Assessment System-second edition; ADOS-2, Autism Diagnostic Observation Scale-second edition; ABaCo, Assessment Battery for Communication.

To define eligibility, at the baseline, each patient carried out a standardized neuropsychiatric and psychodiagnostic assessment of the cognitive and adaptive skills, the severity of autistic symptoms, and the pragmatic and conversational level of abilities. After the screening, all participants were randomly assigned in a 1:1 ratio to two different groups, which differed only in the order of tDCS delivery: (1) “active-sham”, consisting of five sessions of active tDCS followed by five sessions of sham tDCS or (2) “sham-active”, consisting of five sessions of sham tDCS followed by five sessions of active tDCS. Thus, each participant underwent a total of 10 treatment sessions, combined with cognitive behavioral-oriented training (CBT) focused on the improvement of pragmatic and conversational skills. Each patient repeated the assessment of the pragmatic and conversational abilities at the end of both active and sham tDCS conditions in order to capture changes specifically induced by active stimulation, independent of the order of administration. The study design is summarized in Figure 1.

### 2.3. Neuropsychiatric Assessment

Cognitive and Adaptive skills assessment

In order to quantify the level of intellectual functioning and adaptive skills, the Wechsler Adult Intelligence Scale-Revised (WAIS-R) [26] and the Adaptive Behavior Assessment System-II (ABAS-II) [27] were used, respectively.

The WAIS-R represents the gold standard for the assessment of the cognitive abilities of individuals aged from 16 to 99 years, providing a measure of a verbal IQ, performance IQ, and a full Intelligence Quotient (IQ) that includes both verbal and non-verbal abilities.

The ABAS-II is a parent-report questionnaire that provides a standardized measure of the individual’s adaptive functioning ranging from 0 to 21 years of age. According to the inclusion criteria of the present study, the “5–21 years” form was administered, tailored to assess the level of independence of young adults aged 18–21 years. The ABAS-II provides a General Composite Score (GAC), representing the global level of the individual level of independence, and three main domains: conceptual adaptive domain (CAD), practical adaptive domain (PAD), and social adaptive domain (SAD).

Both WAIS-R and ABAS-II provide composite scores standardized with a mean of 100 and a standard deviation of 15.

Autism symptoms severity assessment

The Adult Diagnostic Observation Schedule-2 (ADOS-2) [28] is a semi-structured observational tool to assess the level of autism symptom severity, providing a total score (TOT) and two subscales according to the DSM-5 diagnostic criteria: Social Affect (AS) and Restricted and Repetitive Behaviors (RRB). The ADOS-2 observation is organized into five modules (Toddler and 1–4 modules), whose administration is based on the chronological age and the individual’s level of expressive language. All the ADOS-2 modules provide a Calibrated Severity Score (CSS) that reflects the severity level of autism symptoms on a scale from 1 to 10.

In the present study, module 4 was administered to all the participants by two certified clinicians (Lucrezia Arturi and Chiara Scoppola, clinical psychologist and child and adolescent psychiatrist) following the ADOS-2 manual, and the revised algorithm for CSS developed by Hus and Lord [29] was used, to quantify the presence of autistic symptoms.

Pragmatic and Communication skills assessment

The Assessment Battery for Communication (ABaCo) [30] is a clinical instrument to evaluate pragmatic skills, intended as the ability to understand and appropriately produce linguistic and extralinguistic acts, depending on the context and social norms.

The battery comprises a total of five scales (linguistic, extralinguistic, paralinguistic, context, and conversation) that—except for the conversation scale—are organized into two major domains, “comprehension” and “production”, providing a total of nine subscales. During the administration of the battery, the individual is asked to set up a short conversation or to watch different videos (20–25 s) and respond to the video-related questions. To each item, a score of 1 (correct) or 0 (incorrect) is assigned, and the sum of the raw scores is converted for each subscale into composite scores and percentiles. The test also provides a global composite score (GLO) reflecting the general level of pragmatic abilities.

In this study, the two alternative and comparable short forms of the battery (form A and B) were used at different time points (baseline, post-active tDCS, post sham, follow-up) to test the efficacy of the treatment and reduce the risk of bias.

### 2.4. Pragmatic Skills Training

Cognitive-behavioral therapy (CBT) is a structured and goal-oriented type of evidence-based psychological intervention aimed at developing individual coping strategies [31].

Different CBT-based trainings have been adapted for the characteristics of people with ASD, demonstrating an efficacy in improving socio-communicative abilities [32].

In the present study, each participant underwent a total of 10 daily sessions (Monday-Friday) of CBT-based training, tailored for the improvement of pragmatic and conversational skills. The training was conducted by a clinical psychologist experienced in evidence-based treatments for individuals with ASD.

Each session of the training was designed to be focused on specific domains of pragmatic language (Table 1). During the sessions, the participant was mainly asked to establish a natural conversation with the therapist, encouraging the appropriate use of both linguistic and paralinguistic forms of communication (i.e., gestures, eye contact, and tone of voice), complete exercises, watch and discuss videos, or take part in role-playing.

### 2.5. Transcranial Direct Current Stimulation (tDCS)

tDCS represents a non-invasive brain stimulation technique able to induce and modulate cortical plasticity via the application of a low-intensity electric current flow (1–2 mA), generated and propagated on the scalp through electrodes covered with small sponges soaked in saline solution [33]. Continuous, constant, and weak currents alter the firing frequency of neurons, which is the neurophysiological mechanism used in tDCS. In particular, the spontaneous firing rate of neurons increases after the application of anodal tDCS (A-tDCS), while the opposite effect is observed after cathodal tDCS (C-tDCS).

In the present study, A-tDCS over the left Broca’s area was applied using a battery-driven Eldith (neuroConn GmbH, Ilmenau, Germany) Programmable Direct Current Stimulator with a pair of surface-soaked sponge electrodes (5 cm × 7 cm).

Active stimulation consisted of 20 min of 1 mA direct current with the anode placed on the left Broca’s area (F5 according to the extended international 10–20 system for electroencephalography—EEG electrode placement) and the cathode over the contralateral supraorbital area.

Sham stimulation was performed with the same electrode montage of active stimulation. In order to mimic the somatosensory artifact of active tDCS, the ramp-up phase was followed by 30 s of stimulation, which was immediately followed by the ramp-down phase. The type of stimulation was triggered by a code number that neither the operator who administered the tDCS nor the subjects participating in the study could identify [34].

Each condition (active and sham) was delivered for all individuals for 5 consecutive days (Monday–Friday), for a total of 10 sessions, interspersed with a 14-day break between the two conditions to minimize carryover effects. The order of the conditions was blindly randomized for both researchers and participants over the duration of the study (4 weeks). Each participant completed a questionnaire on the side effects of tDCS at the end of each experimental session (see Fertonani et al.) [35]. All participants well tolerated the stimulation, reporting mild and transitory tingling in the active electrode site area. No major side effects were reported.

### 2.6. Statistical Analyses

Statistical evaluations were performed using IBM SPSS Statistics for Windows, Version 22.0 [36].

A repeated-measures analysis of variance (ANOVA) was run for each of the composite scores of the 5 subscales of the ABaCo battery.

For each analysis, two within-subject factors were included: *Time* (baseline (T0) vs. end of treatment (T1)) and *Condition* (active tDCS vs. Sham). The interaction was explored using Bonferroni’s post hoc test.

Further analyses were performed in order to correlate clinical features at the baseline and pragmatic skills measures.

## 3. Results

### 3.1. Baseline Demographic and Clinical Features

A final sample of 10 right-handed young adults diagnosed with ASD was included after completing all the sessions of the study protocol (Table 2).

All participants included were males, and the mean age was 19.4 ± 1.83 years (age range 18–25).

At baseline (T0), mean IQ score was 89 ± 20.5, and the mean general adaptive skills (ABAS-II GAC) was 85.1 ± 13.8. Focusing on autistic symptoms, the ADOS-2 CSS mean score was 6 ± 2.1 (moderate level of symptoms’ severity). In terms of communicative and pragmatic skills, the ABaCo GLO mean score was 65.5 ± 11.7, thus, highlighting a severe global impairment of these abilities in the whole group.

### 3.2. Post-Treatment Changes in Pragmatic and Communicative Skills

**Figure 2:**
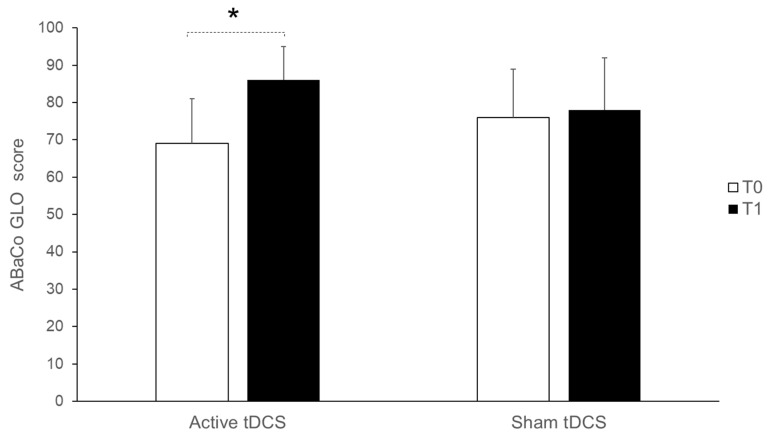
Changes in pragmatic and communicative skills following active or sham tDCS. ABaCo GLO at baseline (T0) and at the end of the treatment (T1) for the active and sham tDCS conditions (* *p* ≤ 0.01). Error bars represent SD.

Our results showed a statistically significant effect of *time* (baseline vs. end of treatment, F_(1,9)_ = 199.77 *p* = 0.002) and interaction *time × condition* (F_(1,9)_ = 62.24; *p* = 0.03) on the overall communicative and pragmatic skills’ improvement assessed by the ABaCo battery. Bonferroni’s post hoc analysis revealed no statistically significant differences in the global pragmatic abilities (GLO) between the two conditions at baseline (T0) (active vs. sham condition = −7; *p* = 1). At the end of the treatment (T1), the mean difference in response accuracy significantly improved only in the active stimulation condition (active stimulation differences between pre-tDCS (T0) vs. post-tDCS (T1) = −17, *p* = 0.005; sham condition differences between pre-sham vs. post-sham = −2; *p* = 1). We therefore ran further analysis, adding the order of condition (active tDCS vs. sham) as a fixed factor. No statistically significant effect came out (F_(1,8)_ = 0.003; *p* = 1) (see Figure 2). In order to estimate a more objective gain attributable to the tDCS intervention while controlling for possible baseline differences between groups, we conducted a non-parametric analysis of covariance (ANCOVA) using Quade’s test with the differential score (post-test minus pre-test) as the dependent variable, group type as the independent variable, and the pre-test as a covariate. The analysis revealed a significant effect of group type (active tDCS vs. sham) on the GLO after adjusting for pre-test performance (Quade’s test, F = 8.169, *p* = 0.01). Pairwise comparisons showed a significant difference between the active tDCS and sham groups, indicating that the active tDCS intervention had a significant impact on GLO changes, independent of baseline performance.

### 3.3. Correlations Between Clinical Features and Pragmatic and Communicative Skills Outcome

At baseline, a negative correlation was found between the verbal IQ domain and ADOS-2 CSS (r = −0.79; *p* = 0.01), while a positive correlation was found between verbal IQ and ABAS-II GAC (r = 0.74; *p* = 0.01). Finally, a negative correlation between ADOS-2 CSS and ABAS-II GAC came out (r = −0.85; *p* = 0.002).

Focusing on communication and pragmatic skills at the baseline, our results showed a statistically significant correlation between ABaCo GLO e verbal IQ domain (r = 0.80; *p* = 0.07) and ABaCo GLO e ABAS-II GAC (r = 0.73; *p* = 0.02).

Particularly regarding the correlation of the baseline clinical phenotype with the pragmatic skills, a negative correlation (r = −0.71; *p* = 0.02) was found between verbal IQ scores and GLO improvement after treatment. Moreover, a negative correlation was found between worse baseline pragmatic performances and their improvement after active tDCS stimulation (ABaCo GLO baseline vs. ABaCo GLO improvement post tDCS: r = −0.67; *p* = 0.04).

Finally, no statistically significant correlations were found between GLO improvement and ADOS-2 CSS (r = 0.37; *p* = 0.29).

## 4. Discussion

To the best of our knowledge, this is the first pilot study specifically aimed at determining whether anodal tDCS delivered over the left Broca’s area combined with cognitive behavioral-oriented training (CBT) could improve pragmatical and conversational abilities in young adults with ASD.

Pragmatic skills and socio-communication impairment are common features in ASD, regardless of age and symptoms’ severity [6,9,37]. Consistent with these findings, our sample showed severe global impairment in communicative and pragmatic skills (ABaCo GLO = 65.5 ± 11.7), despite an adequate level of intellectual functioning (WAIS-R total = 89.0 ± 20.5) and a moderate level of autism symptom severity (ADOS-2 CSS = 6).

The application of tDCS together with CBT training, in the present study, determined a significant improvement in global pragmatic abilities after active stimulation compared to sham. Our results align with previous research reporting improved communication abilities after anodic tDCS, applied over the left Broca’s region, and intensive conversational treatment in other neurological conditions [22].

As a matter of fact, the activation of Broca’s area during tasks involved in pragmatic competencies was found to be crucial in studies involving neurofunctional and electrophysiological tools (near-infrared spectroscopy-NIRS and event-related potentials-ERPs) [24,38,39].

In addition, the modulator effect of the tDCS on the Broca’s area may facilitate brain connectivity and synaptic plasticity to other regions interconnected with Broca, such as the prefrontal cortex, which in turn could contribute to the improvement of executive functions, ToM, and pragmatic language abilities, resulting in enhancement understanding context and using appropriate language according to social situations [6,22,24,40,41,42]. Our results are coherent with this interpretation, suggesting that also in ASD, Broca’s area represents a cortical region crucial for the processes through which language is contextualized.

Furthermore, our results seem to suggest the potential role of the tDCS as an additional boost to facilitate the socio-pragmatic skills acquisition induced by the CBT itself. Indeed, even if tDCS per se represents a promising therapeutic option in the context of different neuropsychiatric conditions, including ASD, previous trials have yet highlighted the superiority of the combination of active tDCS with psychological and/or pharmacological treatments [17,18].

Moreover, our results showed a negative correlation between baseline ABaCo scores, verbal IQ levels, and pragmatic skills improvement after the treatment, indicating that autistic individuals with greater communicative skills impairment (ABaCo scores and WAIS-R verbal IQ) responded better to the treatment program. This result is in line with previous tDCS studies conducted in the context of verbal language treatment, demonstrating the effectiveness of tDCS treatment in individuals with more severe language and cognitive impairment at the baseline due to several interrelated factors [43,44].

One explanation of this phenomenon stems from the hypothesis that those individuals with more severe deficits may develop compensatory processes that, in turn, lead to an enhancement of neuroplasticity mechanisms. This enhancement may further facilitate the more effective reorganization of neural pathways in response to stimulation [33]. Additionally, it can be speculated that the larger the functional gap between baseline and post-treatment performances, the more notable the improvements in daily functioning and their evaluation through standardized tools. Moreover, individuals with higher performance at baseline may also have more stable overall performance associated with higher global competence. This could further complicate the assessment of pre- and post-treatment improvements with tDCS [43].

Finally, our results did not show statistically significant correlations between global pragmatic and communicative improvement and the severity level of autistic symptoms. This result could be associated with several aspects; firstly, the severity of autistic symptoms may result from different factors (e.g., cognitive, adaptive skills), reflecting individual variability and the heterogeneity of ASD phenotype [45]. Furthermore, it should be taken into account that the overall severity of autistic symptoms is not exclusively attributable to the impairment of the socio-relational domain—including pragmatic communication—but also to the presence of restricted interests, repetitive behaviors, and atypical sensory sensitivity. Additionally, our results align with the existing literature suggesting that the impairment of pragmatic and communicative abilities in autistic individuals is more closely linked to the lack of ToM and the deficit of executive functioning (e.g., problem-solving, set shifting) rather than the overall severity of symptoms [6,46,47,48]. According to this assumption, and since Broca’s area is involved in executive control [6,24,49,50,51], it is possible that the stimulation of this brain region via tDCS, combined with targeted training, leads to the enhancement of the global communication competencies.

Unfortunately, the lack of available data on the topic does not allow us to compare our results to findings from other neuromodulation studies. Therefore, studies specifically aimed at understanding the potential role of tDCS in the socio-pragmatic skills of ASD individuals are needed.

Indeed, the improvement of communicative and pragmatic abilities plays a pivotal role in the enhancement of overall social functioning [10]; thus, the application of tDCS as an add-on to traditional interventions should be taken into consideration to improve the efficacy of the rehabilitation treatments in clinical settings given the feasibility, safeness, and cost-effectiveness of the technique [52,53].

Overall, our study presents many strengths and useful clinical implications. Firstly, the clinical evaluations carried out at each time point of the present study were conducted using standardized tools directly administered by clinicians to minimize the heterogeneity within the sample and/or to limit the risk of bias induced by the parent- or self-report measures commonly used in the field of psychiatry research. Moreover, the concurrent delivery of tDCS and CBT represents a crucial methodology for examining the cumulative effects of different interventions and exploring portable and cost-effective treatment strategies leading to the improvement of autistic individuals’ QoL.

Nonetheless, despite these strengths, our results should be interpreted with caution due to the limitations of the study. As a matter of fact, the effect of the active tDCS in comparison to the sham was slight, probably due to the small sample size and the limited number of CBT training sessions. In addition, the trial was restricted only to autistic individuals without severe cognitive and verbal impairment. For this reason, and due to ASD clinical heterogeneity, our findings cannot be generalizable to the entire ASD population and, in this regard, further studies including autistic individuals with more severe clinical manifestations and, importantly, larger sample sizes are needed.

## 5. Conclusions

In conclusion, the preliminary findings of our pilot study confirm that the application of anodal tDCS is safe and feasible. Despite the small sample size, the ABaCo GLO changes indicate superior effectiveness of tDCS on Broca’s area as an add-on to CBT training focusing on communicative and pragmatic skills. The improvement of communicative and pragmatic abilities plays a pivotal role in the enhancement of overall social functioning [10,54]; thus, the application of tDCS as an add-on to traditional interventions should be taken into consideration to improve the efficacy of the rehabilitation treatments.

From a clinical point of view, the positive effect of the treatments’ combination was observed regardless of autism symptoms’ severity, suggesting that interventions targeting neural circuits involved in pragmatic language (such as Broca’s area) may be useful and generalizable across the spectrum, contributing to improving individuals’ QoL.

## Figures and Tables

**Table 1:** Main activities and topics of the CBT sessions.

Sessions	Domain	Tools and Techniques	Main Topic
day 1, 5, 6, and 10	Conversational	Free conversation	Adherence to the topic
			Respect of turn-taking
			Modulation of eye-contact
			Appropriate use of gestures and tone of voice
day 2 and 7	Extralinguistic	Comic strip conversation, social stories, role playing	Comprehension and production of ironic sentences
			Comprehension and production of deception sentences
day 3 and 8	Paralinguistic	Social stories, role playing, video	Comprehension and production of communication acts expressing emotions
			Comprehension and production of acts characterized by a paralinguistic contradiction
day 4 and 9	Linguistics/ Context	Social stories, role playing	Comprehension and production of statements, answers, requests, and commands
			Adherence to discourse norms
			Social adequacy in different context

**Table 2:** Demographic and clinical characteristics at the baseline evaluation.

**Age**	19.4 ± 1.83
**Sex ratio (male/female)**	10/0
**Full IQ**	89.0 ± 20.5
Verbal IQ	90.8 ± 24.0
Performance IQ	95.1 ± 26.4
**ABAS-II**	
ABAS-II GAC	85.1 ± 13.8
ABAS-II CAD	89.3 ± 13.9
ABAS-II SAD	81.5 ± 9.6
ABAS-II PAD	85.1 ± 16.8
**ADOS-2**	
ADOS-2 SA	9.4 ± 3.8
ADOS-2 RRB	2 ± 1.4
ADOS-2 CSS	6 ± 2.1
**ABaCo GLO**	65.5 ± 11.7

Note: all the scores in the table are reported in means and standard deviations and refer to the Intelligence Quotient (IQ); Adaptive Behavior Assessment System second edition (ABAS-II) with the General Adaptive Composite (GAC), conceptual (CAD), practical (PAD), and social (SAD) domains; Autism Diagnostic Observation Schedule second edition (ADOS-2) with Social Affect (SA), Restricted and Repetitive Behaviors (RRB), and Calibrated Severity Score (CSS); and global score of the Assessment Battery for Communication (ABaCo GLO).

## Data Availability

The data presented in this study are contained within the article.
